# Acute exposure to silica nanoparticles enhances mortality and increases lung permeability in a mouse model of *Pseudomonas aeruginosa* pneumonia

**DOI:** 10.1186/s12989-014-0078-9

**Published:** 2015-01-21

**Authors:** Mathilde Delaval, Sonja Boland, Brigitte Solhonne, Marie-Anne Nicola, Stéphane Mornet, Armelle Baeza-Squiban, Jean-Michel Sallenave, Ignacio Garcia-Verdugo

**Affiliations:** Univ Paris Diderot. Sorbone Paris Cité. Unit of Functional and Adaptive Biology (BFA) UMR 8251, CNRS, Laboratory of Molecular and Cellular Responses to Xenobiotics, 5 rue Thomas Mann, 75013 Paris, France; Unité de Défense Innée et Inflammation, Institut Pasteur, 25 rue du Dr Roux, 75015 Paris, France; INSERM U874, Institut Pasteur, 25 rue du Dr Roux, 75015 Paris, France; INSERM U1152, Faculté de Médicine site Bichat, Université Paris Diderot, 16, rue Henri Huchard, 75018 Paris, France; Plateforme d’imagerie dynamique, Institut Pasteur, 25 rue du Dr Roux, 75015 Paris, France; CNRS, Univ. Bordeaux, ICMCB, UPR 9048, 87 Avenue du Docteur A. Schweitzer, Pessac cedex, F-33600 France; Université Sorbonne Paris Cité, Cellule Pasteur, Université Paris Diderot, rue du Dr Roux, 75015 Paris, France

**Keywords:** SiO_2_, Nanoparticles, *Pseudomonas*, Lung, Inflammation, Infection, Alveolar macrophages, Alveolar permeability

## Abstract

**Background:**

The lung epithelium constitutes the first barrier against invading pathogens and also a major surface potentially exposed to nanoparticles. In order to ensure and preserve lung epithelial barrier function, the alveolar compartment possesses local defence mechanisms that are able to control bacterial infection. For instance, alveolar macrophages are professional phagocytic cells that engulf bacteria and environmental contaminants (including nanoparticles) and secrete pro-inflammatory cytokines to effectively eliminate the invading bacteria/contaminants. The consequences of nanoparticle exposure in the context of lung infection have not been studied in detail. Previous reports have shown that sequential lung exposure to nanoparticles and bacteria may impair bacterial clearance resulting in increased lung bacterial loads, associated with a reduction in the phagocytic capacity of alveolar macrophages.

**Results:**

Here we have studied the consequences of SiO_2_ nanoparticle exposure on *Pseudomonas aeruginosa* clearance, *Pseudomonas aeruginosa*-induced inflammation and lung injury in a mouse model of acute pneumonia. We observed that pre-exposure to SiO_2_ nanoparticles increased mice susceptibility to lethal pneumonia but did not modify lung clearance of a bioluminescent *Pseudomonas aeruginosa* strain. Furthermore, internalisation of SiO_2_ nanoparticles by primary alveolar macrophages did not reduce the capacity of the cells to clear *Pseudomonas aeruginosa*. In our murine model, SiO_2_ nanoparticle pre-exposure preferentially enhanced *Pseudomonas aeruginosa*-induced lung permeability (the latter assessed by the measurement of alveolar albumin and IgM concentrations) rather than contributing to *Pseudomonas aeruginosa*-induced lung inflammation (as measured by leukocyte recruitment and cytokine concentration in the alveolar compartment).

**Conclusions:**

We show that pre-exposure to SiO_2_ nanoparticles increases mice susceptibility to lethal pneumonia but independently of macrophage phagocytic function. The deleterious effects of SiO_2_ nanoparticle exposure during *Pseudomonas aeruginosa*-induced pneumonia are related to alterations of the alveolar-capillary barrier rather than to modulation of the inflammatory responses.

**Electronic supplementary material:**

The online version of this article (doi:10.1186/s12989-014-0078-9) contains supplementary material, which is available to authorized users.

## Background

The explosion in the use of manufactured nanoparticles (NPs) will undoubtedly be associated worldwide with a potentially increased exposure (accidental or intentional) of the population to these agents. The lung epithelium constitutes the largest surface of contact with the environment, and as such, can be considered as the major surface accidentally exposed to NPs. However, the lung epithelium should also be regarded as a desired target for the delivery of drugs or diagnostic agents based on NPs (intentional exposure). Among NPs, SiO_2_ NPs are produced in high quantities and have important applications not only in consumer products but also in the field of nanomedicine [1].

Once inhaled, NPs will be deposited in different regions of the lungs, including the alveoli [2]. After their entry in the alveolar space, NPs encounter alveolar macrophages (AMs) and epithelial cells. AMs are professional phagocytic cells that recognize invading microbes, including bacteria, and release cytokines and inflammatory mediators needed to mount host responses against these pathogens [3]. Bacterial clearance by AMs is initiated by recognition of the pathogen by phagocytic receptors at the cell surface. Then, further internalization occurs, ultimately resulting in the degradation of the engulfed bacteria in the phagolysosome [4].

Particularly relevant to the lung, *Pseudomonas aeruginosa* (*P.a*) can be found in ventilator-associated pneumonia in nosocomial infections [5]. Importantly, it also colonizes the lungs of cystic fibrosis patients contributing significantly to morbidity and mortality in this disease [6]. *P.a* respiratory infection is associated with lung inflammation and increased permeability of the alveolocapillary barrier [7]. Eradication of *P.a* from the lungs of the patients is difficult due to multiple drug-resistance of the bacterium. In that context, new promising therapies may be based on the use of silica-derived NPs as efficient carriers for antibacterial agents [8,9]. However in order to develop a therapeutic use of NPs against lung infections, it is important first to characterise the consequences of NP exposure on target cells, especially innate immune cells. Indeed inhaled NPs can target immune cells, including AMs. Uptake of NPs by AMs might follow endocytic as well as non-endocytic pathways, including passive diffusion [10,11]. Once they are engulfed, NPs can accumulate in lysosomes of macrophages [12-14]. Considering that bacterial and NP uptake converge within the endocytic pathway, it is rational to hypothesize that adverse effects of NP exposure might include impairment of phagocyte function. Indeed it has been shown that *in vitro* exposure of macrophages to carboxyl polystyrene or aluminium derived NPs hindered their phagocytic capacity [15,16]. Moreover, intra-tracheal instillation of small-sized titanium dioxide to rats modified the phagocytic capacity of isolated AMs in a concentration dependent-manner [17]. In the context of lung infection, recent studies indicate that sequential lung exposure to carbon nanotubes or cupper NPs followed by *Listeria monocytogenes* or *Klebsiella pneumoniae* challenge, respectively increase lung inflammation and reduce bacterial clearance compared with animals not exposed to NPs [18,19]. Although impaired bacterial clearance was associated with reduced phagocytic activity of AMs after NP exposure [19], neither of these studies could discriminate the effects of NPs alone, from the effects of NPs plus bacteria, since NPs alone administration was associated with inflammation and lung injury. In consequence, effects of NP pre-exposure on lung infection could be due to NP-associated inflammation rather than to a direct action on AM phagocytic activity.

Here we have evaluated the effects of SiO_2_ NP pre-exposure followed by *P.a* infection, two environmental insults not studied in combination before. We focussed our study in determining the consequences of SiO_2_ NP exposure on *P.a* clearance and *P.a*-induced inflammation and lung injury in a mouse model of acute pneumonia. We selected short exposure times in order to reduce lung inflammation potentially associated with NPs and to optimize the uptake of SiO_2_ NPs by AM *in vivo*. We report here that pre-exposure to SiO_2_ NPs increased mice susceptibility to lethal pneumonia but interestingly independently of an impairment of AM phagocytic function. Instead, SiO_2_ NPs pre-exposure enhanced *P.a*-induced alterations of the lung alveolar-capillary barrier without affecting lung inflammation.

## Results

### Characterisation of silica NPs

Fluorescent or unlabelled 15 nm SiO_2_ NPs were synthetized and characterized by dynamic light scattering (DLS) as previously described [20,21]. DLS analysis demonstrated that both types of NPs presented a hydrodynamic diameter of 44.4 and 50.9 nm respectively in water (Table 1) showing a low aggregation rate in solution. Moreover, NP preparations were relatively stable in physiological media because the hydrodynamic diameter did not change when increasing ionic strength of the media (compare hydrodynamic diameter of NP suspensions in water versus PBS or RPMI culture media). In addition we measured endotoxin content in NP preparations by the LAL method [22] (Table 1). Following this procedure the quantity of endotoxin detected in our NP preparations was <0.25 EU/mg. These measurements were performed at doses of NPs that did not interfere with the LAL chromogenic test.

**Table 1:** **Nanoparticle characterisation**

**Nanopaticle**	**Physico-chemical properties**									**Endotoxin**
	**Water**			**PBS**			**RPM**			
	**Size (nm)**	**Pdl**	**Z-pot (mV)**	**Size (nm)**	**Pdl**	**Z-pot (mV)**	**Size (nm)**	**Pdl**	**Z-pot (mV)**	
SiO_2_	50.9	0.271	−11.2	53.6	0.217	−10.1	68.1	0.207	−13.2	<0.25 EU/mg
FITC-SiO_2_	44.4	0.262	−64.5	56.4	0.306.	−10.5	54.4	0.305	−21.9	<0.25 EU/mg

Labelled or unlabelled 15 nm SiO2 NPs were characterised in different media. The hydrodynamic size of agglomerates and polydispersity index (PdI) of suspensions were determined by DLS and their charge by measuring the zeta-potential (Z-pot). Endotoxin detection was performed by the *Limulus* amebocyte Assay (LAL) method following manufacturer’s instructions. Detection limit of the assay was 0.1 EU/ml and concentration of NPs in the assay was 0.4 mg/ml.

### Silica NPs do not induce lung inflammation 5 h after instillation and colocalize with alveolar epithelial cells at longer exposure time

**Figure 1:**
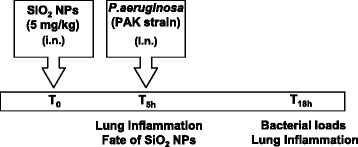
**Experimental design.** C57Bl/6j mice were intranasally instilled with silica nanoparticles (5 mg/Kg) or vehicle and 5 h post NPs instillation infected with *P.aeruginosa* by intranasal instillation. 18 h after infection, inflammatory markers in the lungs were analysed by measuring cytokines and neutrophil recruitment in the broncho-alveolar lavages (BALs). Bacterial loads were studied by bioluminescence after infection with a Pa-luciferase producing strain. Distribution of fluorescently labelled FITC SiO_2_ NPs (Fate of NPs) was followed by immunofluorescence analysis of cells present in BALs and lung tissues.

**Figure 2:**
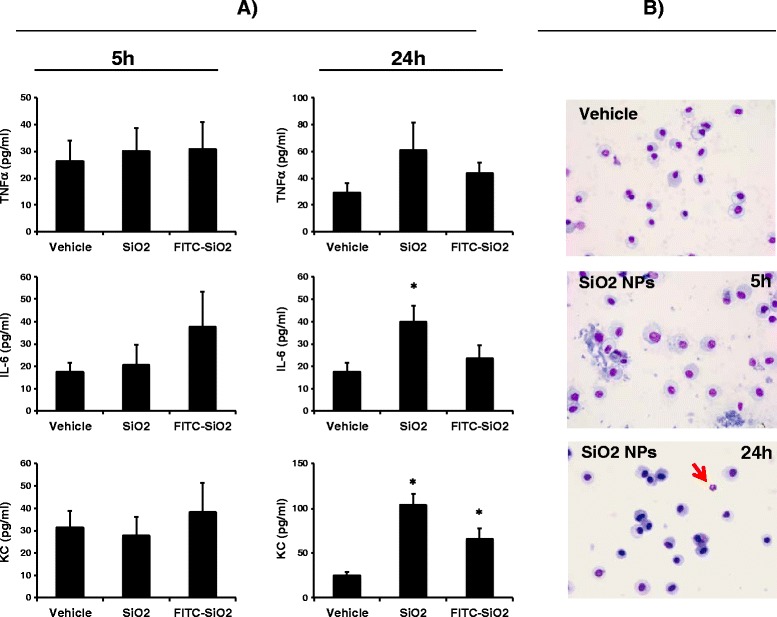
**Effects of silica nanoparticles on lung inflammation after instillation. A)** C57Bl/6j mice were intranasally instilled with FITC-SiO_2_ or unlabelled SiO_2_ nanoparticles (5 mg/kg;100 μg/mice) or vehicle. 5 h or 24 h after, concentration of pro-inflammatory cytokines were measured in BALs (2 ml) (left panels). **B)** Cells present in BALs were cytospined and stained with Diff-Quick to identify macrophages and neutrophils. A representative image of BAL cells in a control mice (upper panel) or SiO_2_ NPs treated mice are shown (lower panels). Neutrophils (red arrow) were found in some NP treated mice 24 h post-instillation and represented less than 10% of total cells in BALs. Mean ± SEM is represented from 4 to 6 mice per group. *p < 0.05 vs. Vehicle, Mann–Whitney test.

**Figure 3:**
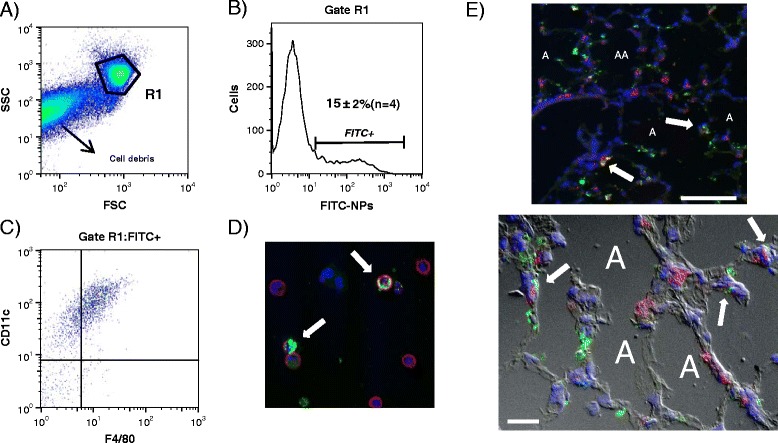
**Silica nanoparticles are internalized by alveolar macrophages and colocalize with alveolar epithelial cells.** C57Bl/6j mice were instilled with FITC-SiO_2_ NPs (5 mg/kg;100 μg/mice). **A)** 5 h later, mice were sacrificed and cells present in BALs were analysed by flow cytometry after labelling with specific antibodies directed against alveolar macrophages markers (CD11c, F4/80). Panel **B)** shows that 15 ± 2% (n = 4, mice) of the cells were FITC positive. Among them, over 95% were CD11c + and 85% CD11c + F4/80+ **(panel C)**. The FACs analysis from one representative BAL from a total of 4 is shown. In **D)** BAL cells were cytospined and stained with anti-mouse CD45 antibody followed by Alexa 664 Fluor (A664) coupled secondary antibody (red) to confirm internalisation of FITC-SiO_2_ NPs (white arrows). **E)** Cryo-sections of lung tissues recovered from mice 24 h after instillation of FITC- SiO_2_ NPs (green) were stained with anti-proSPC antibody (a marker of alveolar type II epithelial cells) followed by A664 coupled secondary antibody (red). White arrows indicate co-localisation of NPs and alveolar epithelial cells. White scale bar = 100 μm or 20 μm (upper and lower panel, respectively). Nuclei were stained with DAPI (blue). Fluorescence of stained cells and tissues were analysed under structured illumination (ApoTome system). Superposed images from FITC, A664, DAPI and differential interferential contrast (**D**, lower panel) filters are shown. The ‘A’ within the figures indicates the alveolar spaces.

To study the consequences of NP exposure on *P.a* lung infection, we set up a protocol where mice were first treated with SiO_2_ NPs (5 mg/kg) and 5 h later infected with the *P.a* strain PAK (Figure 1). For most of our experiments, we selected 5 h time after NP treatment as a time point for infection because at that time, we did not observed obvious signs of inflammation (including neutrophil accumulation) in the lungs of NP treated mice compared to controls (Figure 2). In addition, because neutrophil influx usually starts 6 hours post-LPS instillation [23,24], this indirectly also confirms that LPS is undetectable and if present, is biologically inactive in our NP preparations. However, in one parallel experiment, 24 h after NP instillation a slight inflammation was detected in the lungs characterized by increased levels of KC (CXCL-1, a neutrophil chemokine) and a slight neutrophil accumulation (less than 10% of total cells) (Figure 2). Higher doses of SiO_2_ NPs increased pro-inflammatory cytokines 24 h after treatment (data not shown). Because our goal was to study NP effects independently of its pro-inflammatory activity, we therefore selected a dose of 5 mg/Kg for the SiO_2_ NP treatments and chose 5 h post NP instillation as the time point when *P.a* was administered (see below). At that time point and in accordance with the quasi-exclusive presence of AM (over 90%) and the absence of neutrophils in BALs, FITC-SiO_2_ or unlabelled SiO_2_ NP treatment did not increase the basal levels of TNFα, KC, or IL-6 in BAL fluid (Figure 2). Interestingly, at 5 h post-NP instillation, over 15% of the cells present in the BAL (mostly AM) internalized FITC-SiO_2_ NPs (Figure 3B and D, white arrows). In accordance with the cytospin analysis (Figure 2) most of the cells that internalized SiO_2_ NPs presented an alveolar macrophage phenotype (CD11c^+^ F4/80^+^) (Figure 3C). Therefore, these data and the absence of inflammation after NP treatment alone allowed us to study the consequences of NP exposure on target cells without the confounding indirect effects of pro-inflammatory response associated with NPs treatment. Indeed, 24 h after FITC-SiO_2_ instillation, cryo-sections of lungs showed that FITC-SiO_2_ NPs were associated with lung epithelium and colocalized with alveolar type II epithelial cells (labelled with an anti-pro surfactant protein-C (SPC) antibody) (Figure 3E). Association of FITC-SiO_2_ with alveolar epithelial cells (AEC) could explain the increased levels of KC (a typically epithelial cell secreted chemokine) found in BALs 24 h post instillation (Figure 2).

### Silica NPs enhance permeability of alveolar-capillary barrier

**Figure 4:**
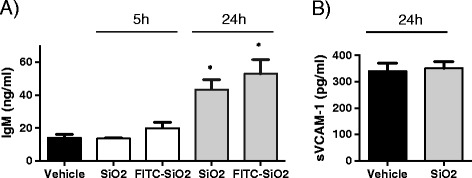
**Effects of silica NPs in alveolar-capillary permeability.** C57Bl/6j mice were instilled with 5 mg/kg (100 μg/mice) of SiO_2_ NPs, FITC- SiO_2_ NPs or vehicle. 5 h or 24 h later, mice were sacrificed and IgM **(A)** and sVCAM-1 **(B)** concentrations in BALs (2 ml) were measured by sandwich ELISA. Mean ± SEM is represented from 5 mice per group *p < 0.05 vs. Vehicle, Mann–Whitney test.

Because SiO_2_ NPs associated with AEC (Figure 3), and as AECs guarantee epithelial barrier integrity [25], we wondered whether SiO_2_ NPs could alter the alveolar-capillary barrier integrity. This was assessed by measuring IgM (a pentameric serum protein with a Mr 900 kDa) concentration in BAL fluids. Increased amounts of IgM in BAL fluids are related with alterations in alveolar-capillary barrier and increased lung permeability as previously described [24,26,27]. As show Figure 4A, SiO_2_ NP instillation increased the concentration of IgM in BALs 24 h post treatment. Interestingly, at this time point, levels of soluble vascular cell adhesion molecule-1 (sVCAM-1) in BALs, a parameter related to endothelium injury and activation, was not modified (Figure 4B). These data indicate that SiO_2_ NPs increase alveolar-capillary barrier with minor alterations of the vascular endothelium.

### Pre-exposure to silica NPs enhances mice mortality during *P. a-induced* pneumonia

**Figure 5:**
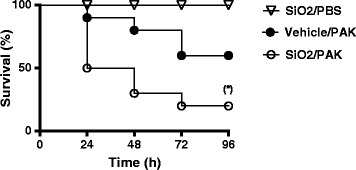
**Survival curves of mice infected with** ***Pseudomonas aeruginosa*** **strain PAK.** C57Bl/6j mice were instilled with 5 mg/kg (100 μg/mice) of SiO_2_ NPs or vehicle. 5 h after, mice were infected with *P. aeruginosa* (PAK strain) (2x10^7^ CFU/mice) and survival was followed over time. (*) p < 0.05 Log-Rank (Mantel-Cox) test (n = 10 mice per group).

Considering that SiO_2_ NPs interact with AM and AEC, two cell types driving lung defence against P.a, we studied the consequences of NP exposure in a mouse model of P.a infection. First we studied the effect of NP exposure on *P.a*-induced mortality in mice. Intranasal administration of 2x10^7^ CFU per mice killed almost 40% of mice 72 h post infection (Figure 5). Crucially, pre-exposure to SiO_2_ NPs 5 h before bacterial challenge enhanced mouse mortality by 20%, indicating a deleterious effect of NP exposure in our mice model of acute pneumonia.

### Pre-exposure to silica NPs does not interfere with bacterial clearance nor bacterial translocation *in vivo*

**Figure 6:**
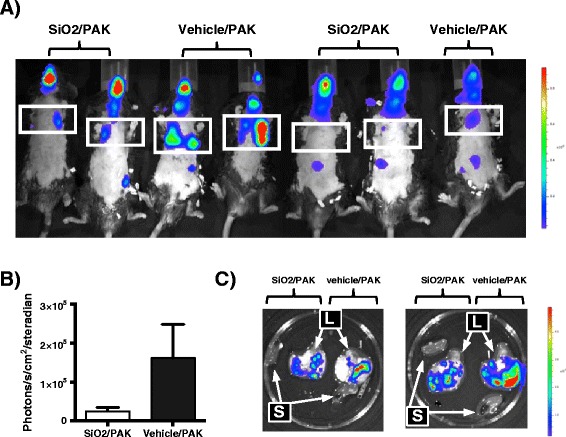
**Effects of silica nanoparticles in bacterial clearance. A)** Mice were treated with silica NPs (SiO2) or vehicle and 5 h later infected with PAK-lux (10^7^ CFU/mice). 15 h after infection, bioluminescence was recorded in the whole animal **(panels A, B)** or in extracted organs **(panel C)**. **B)** Quantification of bioluminescence in the lungs. S = spleen, L = lungs. Colour scale represents photons/s/cm2/steradian. In panel **A)** the rib cage is delimited within the squares.

Our laboratory and others have reported that increased mortality during *P. aeruginosa* pneumonia could be associated with increased bacterial burden [28-30]. We therefore analysed whether SiO_2_ NP pre-exposure enhanced bacterial loads in the lungs of infected mice. To that aim, we studied the elimination of *P.a* in the whole animal using a bioluminescence approach. Mice were therefore infected with 10^7^ CFU of a bioluminescent *P.a* strain (PAK-lux) as previously described [29] and 15 h later, bioluminescence was measured. We observed that NP treated mice did not present a higher bacterial load in lungs, when compared to non-treated mice (Figure 6A and B). Rather NP-treatment showed a tendency to reduce bacterial loads in lungs. To study further whether NP treatment could induce bacterial translocation, we dissected spleen and lungs to analyse associated bioluminescence. As shown in (Figure 6C), we did not detect bacterial growth in the spleen. Analysis of dissected lungs also confirmed that NP exposure did not increase bacterial loads in lungs (Figure 6C).

### Pre-exposure to silica NPs does not interfere with AM-mediated bacterial clearance or AM cytokine output

**Figure 7:**
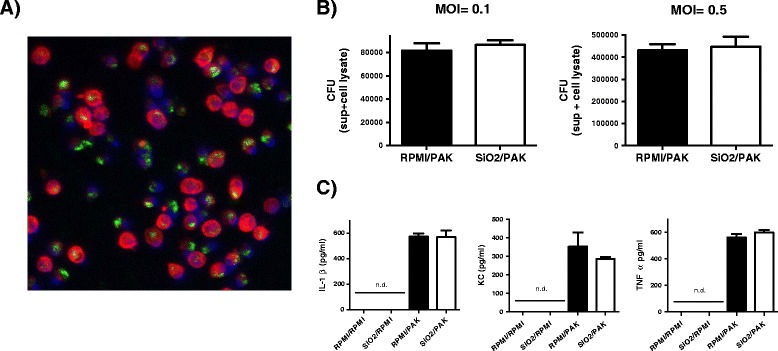
**Effects of silica nanoparticles on bacterial clearance and AM inflammatory responses induced by** ***P.a*** **.** Mice primary AMs (10^5^ cells/well) from BALs were treated ex-vivo with FITC-SiO_2_ **(A)** or unlabelled SiO_2_ NPs **(B and** **C)** for 5 h at 2.5 μg/cm^2^ (0.8 μg/well) in culture media (RPMI) (200 μl). In selected experiments, FITC-SiO_2_ treated cells were fixed and cytoskeleton and nuclei stained with phalloidin–tetramethylrhodamine (red) and DAPI (blue), respectively. Fluorescence images were collected from observation on Apotome microscope **(A)**. After incubation with NPs, supernatants were removed and cells (10^5^ cells/well) challenged with PAK at MOI 0.1 (**B** left panel) or MOI 0.5 (**B** right panel and **C**). After 4 h, supernatants were collected and cells were lysed in 0,1% triton X-100, conditions that allow bacterial growth. Bacterial CFU were counted in cell lysates and supernatants (CFU super + cell lysate) as shown in **B**. Concentration of cytokines were measured in cells supernatants (200 μl) from untreated cells (RPMI/RPMI), cells treated with SiO_2_ NPs (SiO2/RPMI), cells challenged with bacteria (RPMI/PAK) and cells exposed to SiO_2_ NPs and then challenged with bacteria (SiO2/PAK). Means ± SEM of triplicates from three independent pools of alveolar macrophages are represented. Experiments were repeated twice. (n.d.) = not detectable.

AMs play a key role in *P.a* elimination from infected lungs [7] and previous data have shown that NP exposure may alter the capacity of AMs to eliminate bacteria [15-17,31]. To definitely rule out a decrease in bacterial clearance following NP treatment, we assessed the effect of NPs on AM killing of *P.a* and on AM cytokine output. *Ex-vivo* experiments on AM were performed by adding FITC or unlabelled SiO_2_ NPs at non-toxic doses (2.5 μg/cm^2^) at t0. 5 h later, over 50% of alveolar macrophages (53 ± 10%, n = 3) had internalized FITC-SiO_2_ NPs (Figure 7A). At that time point, extracellular NPs were washed out and macrophages challenged with *P.a* (MOI = multiplicity of infection, 0.1 and 0.5). 4 h post-infection we measured CFU in supernatants and cellular lysates to evaluate bacterial clearance as previously described [32]. As shown in Figure 7B no differences were observed in the total number of bacteria recovered from alveolar macrophages cell cultures, whether NP had been used or not.

Furthermore SiO_2_ NPs did not modify the *P.a*-induced secretion of KC, TNFα or IL1β by AM (Figure 7C). Taken together these data indicate that internalisation of SiO_2_ NPs by AMs did not modify their capacity to eliminate *P.a* and did not modulate either the inflammatory response induced by this bacterium.

### Pre-exposure to silica NPs enhances alveolar-capillary barrier permeability following *P.a* infection

**Figure 8:**
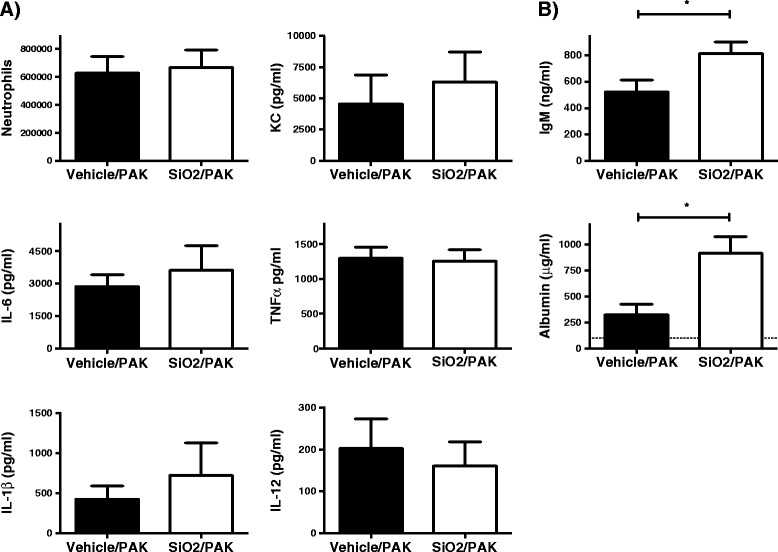
**Effects of silica nanoparticles on inflammatory markers and alveolar-capillar permeability alterations induced by** ***P.a*** **infection.** Mice were treated with SiO_2_ NPs (5 mg/kg;100 μg/mice) (open bar) or vehicle (close bar) and 5 h later infected with PAK (2.5x10^6^ CFU/mice). 18 h after infection, number of neutrophils and concentration of cytokines **(A)**, IgM and albumin **(B)** were measured in BALs (2 ml). Basal levels of KC, IL-6 TNFα in non-infected mice were 31 ± 7, 18 ± 4 and 26 ± 7 pg/ml (n = 4), respectively (see Figure 2). Basal levels of IL-12 and IL-1β were 50 ± 10 pg/ml and not detectable, respectively. Basal levels of IgM in BALs from non-infected mice were 17 ± 4 ng/ml (n = 5) (Figure 4). The dotted line in B) represents basal levels of albumin in non-infected mice. Mean ± SEM is represented from 7 to 8 mice per group, *p < 0.05 Mann–Whitney test.

Because changes in lung bacterial loads and the activation of AM could not explain the detrimental effects of NP exposure on *P.a* infection, we explored whether SiO_2_ NPs may act through different mechanisms. For this purpose, we measured the recruitment of neutrophils and the concentrations of pro-inflammatory cytokines in BAL fluids of mice treated (or not) with NPs and infected with *P.a*. Expectedly, infection of *P.a* (2.5×10^6^ CFU/mice), resulted in a sharp increase in neutrophils associated with increased levels of KC, IL-6, TNFα, IL-1β and IL-12 in BAL fluids (Figure 8) compared to non-infected mice (Figure 2). Importantly, Figure 8A shows that pre-exposure to SiO_2_ NPs did not significantly modify the number of recruited neutrophils post *P.a* infection and neither the concentration of KC, TNFα, IL-6, IL1β or IL-12 in BAL fluids. Finally and in agreement with the capacity of NPs to alter alveolar-capillary barrier (Figure 4), we wondered whether SiO_2_ NPs could enhance the damage to the alveolar-capillary barrier during infection, potentially explaining the observed compromised mice survival (Figure 5). This was assessed by measuring IgM and albumin (another serum protein with a Mr of 67 kDa) concentration in BAL fluids. Remarkably, we observed that IgM and albumin concentrations were increased in the BALs of infected mice pre-exposed to SiO_2_ NP (Figure 8B), potentially explaining the increased susceptibility associated with NP treatment (Figure 5).

## Discussion

Toxicity associated with NP exposure has been widely documented. For instance, it has been described that *in vitro* exposure of macrophages and epithelial cells to NPs resulted in the induction of inflammatory responses including release of pro-inflammatory cytokines and induction of oxidative stress [33-35]. However these effects may depend on the composition of NPs, their physico-chemical properties, presence of impurities as well as on the duration of the exposure [35,36]. Our silica NP preparations showed a relatively low pro-inflammatory activity *in vitro* and *in vivo*. Indeed, 5 h after intranasal instillation of 5 mg/kg of SiO_2_ NP in mice, we did not observe major signs of inflammation in the lungs even when 15% of the cells in BALs (mostly macrophages) had internalized those NPs. This is in stark contrast with classical pro-inflammatory molecules like bacterial lipopolysaccharide (LPS). Inhalation of LPS in mice results in acute lung inflammation characterized by elevated levels of pro-inflammatory cytokines in BAL fluid as soon as 3 h after administration [23]. When mice BAL fluid were analysed 24 h after NP treatment, a very modest inflammation was induced characterized by a low percentage of neutrophils among BAL cells (less than 10%) and a slight increase in pro-inflammatory cytokines (Figure 2). Furthermore, incubation *ex-vivo* of freshly isolated AMs with silica NPs did not induce the release of inflammasome-dependent (IL-1β) or -independent cytokines (TNFα, KC, IL-6, CCL5, IL-12, IL-10, INFγ) after 5 h of incubation (data not shown). In contrast to many previous studies our NPs are well characterized, they are not much aggregated and bacterial LPS was undetectable, probably explaining the very weak inflammatory effect observed.

Several studies have described the consequences of NP exposure on immune cells in terms of cellular activation [36,37]. However fewer have focused on the effects of NPs on subsequent anti-microbial host defence mechanisms. We show here that acute exposure to SiO_2_ NPs increased susceptibility to lethal lung pneumonia induced by *P.a*. Furthermore, effects of NP exposure on bacterial clearance were minimal in contrast to previous reports which show that sequential lung exposure to NPs and bacteria impairs lung bacterial clearance [18,19]. For instance, Kim and co-workers showed that instillation of Cu NPs (from 0.12 to 4 mg/kg) 24 h before infection, increased the number of *K.pneumoniae* found in BALs of infected mice [18]. In another independent study, Shvedova and co-workers found that carbon nanotube pre-exposure (2 mg/kg, 3 days before infection) significantly decreased the pulmonary bacterial clearance of *Listeria monocytogenes* at days 3 and 7 post-infection despite robust inflammatory responses [19]. In contrast to our study, Cu NPs and carbon nanotubes induced significant inflammation ‘per se’, which may have impair bacterial clearance by mechanisms independent of AM phagocytic activity. In addition Shvedova and co-workers have shown that pre-exposure of AMs to carbon nanotubes reduces their phagocytic/killing capacity toward *L.monocytogenes* [19]. By contrast, we show here that pre-exposure of AM to SiO_2_ NPs did not modify *P.a* killing nor *P.a*-mediated cytokine release by AM. Discrepancies between our data and others could be due to the composition of NPs, size, shape, concentration, exposure times or the cell type used [15,17,19,31]. In line with our data, Witasp and co-workers showed that internalisation of mesoporous silica particles (>100 nm of diameter) did not alter the capacity of human-monocyte derived macrophages to engulf apoptotic cells [38].

*P.a* respiratory infection is associated with lung inflammation and increased permeability of alveolocapillary barrier [39,40]. Once in the host, the bacterium activates alveolar resident cells (AM and AEC) to produce chemokines and cytokines [41] that activate immune cells and attract inflammatory cells, including neutrophils, to the alveolar space. Uncontrolled lung inflammation can result in increased tissue damage and rupture of the epithelial barrier integrity with fatal consequences for the host (compromised gas-exchange). To measure the impact of SiO_2_ NP exposure on inflammation and lung permeability we measured the BAL content in neutrophils and pro-inflammatory cytokines as well as albumin and IgM concentration, the two latter reflecting increased lung permeability. Whereas concentration of pro-inflammatory cytokines or neutrophil influx in response to *P.a* infection were not different between SiO_2_ NP exposed or unexposed mice, concentration of albumin and IgM was higher in BAL fluids from the former group. Interestingly, and echoing our findings that NPs do no alter necessarily inflammatory parameters, Swedin and co-workers found recently that pre-exposure to carbon nanotubes did not alter lung inflammatory markers associated with a protozoan parasite infection (*Toxoplasma gondii*) [42].

Although the molecular underlying mechanisms are still unclear, we believe that increased local lung permeability and oedema explain the major susceptibility to lethal *P.a* infection associated with SiO_2_ NP pre-exposure. Several data support this hypothesis : firstly we observed that SiO_2_ NPs target alveolar epithelium 24 h post instillation (Figure 3E) and that NP instillation alone disrupted the alveolar capillary barrier (as assessed by IgM leakage, Figure 4). Further we estimated that less than 1% of instilled NPs translocate from lung into circulation (not shown). Secondly, no systemic effect of NP treatment was apparent, as demonstrated by the absence of P.a translocation into the blood stream and the spleen (Figure 6) and the absence of serum markers of systemic tissue injury (Alanine Aminotransferase and Creatine Kinase activities), 24 or 48 h post-infection (Additional file 1: Figure S1).

Potentially explaining some of our results, oxidative stress has been shown to be a key component mediating *P.a* induced host-responses and lung injury [39,40]. Indeed, recent data indicate that regulation of inflammation and lung permeability could be controlled by different molecular mechanisms. Fu and co-workers have shown that down-regulation of NADPH-oxidase 4 (NOX4) attenuated *P.a*–mediated lung permeability, without affecting inflammatory responses of infected mice. Conversely, knockdown of NOX2 reduced *P.a*–induced cytokine output without altering lung permeability [39]. These data support the concept that lung permeability and inflammation could be regulated by independent mechanisms and therefore that SiO_2_ NPs could preferentially affect permeability rather than inflammation during *P.a* infection. In line with this, it has been recently shown that TiO_2_ and SiO_2_ NPs increased endothelial cell leakiness through physical interactions with the endothelial junction protein VE-cadherin and independently of the activation of pro-inflammatory pathways [43]. These results are in line with our data and point towards a new mechanism of NP toxicity based on an increase of the permeability of epithelial/endothelial barriers and the induction of oedema.

## Conclusions

We show here that pre-exposure to SiO_2_ NPs increases susceptibility of mice to a lethal dose of *P.a*- in an acute model of pneumonia, independently of AM phagocytic function. SiO_2_ NP pre-exposure enhanced *P.a*-induced alveolar permeability and oedema, rather than *P.a*-induced inflammation. An increase in alveolar-capillary permeability is strongly correlated to lung injury and to an impaired gas-exchange, which could explain the increased mortality observed.

## Methods

### Synthesis and characterisation of NPs

15 nm FITC or unlabelled SiO_2_ NPs were synthesized as described previously [20,21] following a slightly modified method described by Van Blaaderen. Briefly, fluorescein isothiocyanate (FITC) was covalently linked to 3-aminopropyl-trimethoxysilane (APS) by reaction of the amino group with the isothiocyanate group. 5 mg of FITC was dissolved in 5 ml of 42.7 mM of APS in ethanol. The fluorescein silane was added to 250 ml of ethanol, 5 ml of TEOS (tetraethyl orthosilicate), 7.6 ml of ammonium hydroxide (28%) and 10.9 ml of water. The reaction was performed for 12 h at 50°C in the dark under magnetic stirring. Estimation of silica concentration in the dispersion was carried out by inductively coupled plasma optical emission spectrometry (ICP-OES) and gravimetric method. DLS values as well as the values of zeta potential of SiO_2_ NPs in different media were measured by Zetasizer (nanoZS, Malvern Instruments, USA). Endotoxin content was measured by the QCL-1000 Endpoint Chromogenic LAL Assay following manufactured instructions (Lonza) at doses of SiO_2_ NPs that did not interfere with the assay. Interference of NPs with the assay was measured by including a fixed quantity of NPs (20 μg) in the tubes containing different concentrations of LPS standard. Optical density at 405 nm of the tubes containing NPs was compared with that of tubes without NPs for the same LPS dose. Considering a detection limit for LAL assay of 0.1 EU/ml, we assumed the LPS contamination level in our NPs preparations to be less than 0.25 EU/mg.

### Animals

7-weeks-old male mice (strain C57BL/6 J) were obtained from Janvier Labs (France). Animals were fed and housed under standard conditions with air filtration and cared for in accordance with Pasteur Institute guidelines and in compliance with European Animal Welfare regulations.

### Bacterial strains

The wild-type strain PAK, a commonly studied *P. aeruginosa* strain, was obtained from S. Lory (Harvard Medical School, Boston, MA), as originally isolated by D. Bradley (Memorial University of Newfoundland, St. John’s, Canada). Luminescent strain of PAK was constructed by inserting luxAB into the neutral att site of the chromosome of strain PAK using a mini-Tn7-lux plasmid provided by Microbiotix, in which luxAB is driven by the lac promoter [44]. Bacteria were grown overnight in Lysogeny Broth (LB) medium at 37°C and then transferred to fresh medium and grown by shaking at 100 rpm for 4–5 h to mid-log phase. The culture was centrifuged at 3.000 × g and the pellet was washed and resuspended in PBS. The OD_600nm_ was adjusted to give the desired inoculum. Inoculum was verified by serial dilutions plated on LB agar to determine the number of colony-forming unit (CFU). Bacterial growth of PAK and PAK-lux strain was shown to be identical [29].

### Co-exposure of mice to NPs SiO_2_ and *P.a*

Mice were anesthetized by intraperitoneal injection of a mixture of ketamine-xylazine, and treated with SiO_2_ NPs (5 mg/kg) (100 μg/mice) diluted in PBS or vehicle (a mixture of PBS and H_2_O) via the intranasal route (50 μL), as described elsewhere [32]. 5 h later, animals were anesthetized and 50 μL of a bacterial suspension containing 0.5 to 2.0 x 10^7^ colony-forming units (CFU) was administered by the intranasal route. In some mice, survival was observed for 1 week after infection, and in others broncho-alveolar lavage (BAL) was performed (2 ml) after pentobarbital euthanasia 18 h after infection. Cell counts were measured in the BAL fluids and cell differential counts (presence of macrophages and neutrophils mainly) were determined after cytospin centrifugation and staining with Diff-Quik products. Number of cells per BAL per mouse are represented.

### Concentration of cytokines, albumin and IgM

Murine cytokine (TNFα, IL-6, KC, IL-12, IL1β) and sVCAM-1 concentrations in BAL fluid were determined using DuoSet sandwich enzyme-linked immunosorbent assay kits (R&D Systems). Cytokines are expressed in pg/ml in BALs (2 ml) present in each mouse for *in vivo* experiments and in pg/ml per well (10^5^ cells/well) in *in vitro* experiments.

Mouse albumin was detected by ELISA sandwich. Briefly: Maxisorb Nunc 96-well plates were coated with capture antibody (goat anti-mouse albumin, 1/500 dilution, Bethyl Laboratories) overnight at 4°C in carbonate/bicarbonate buffer (Sigma). After blocking (5% skim milk-PBS, 1 h 37°C), serial dilutions of BAL fluid or mouse albumin standard (Sigma) in 1% skim milk-PBS were added to the plate and plates were incubated at 37°C for 1 h. After washing, secondary antibody (goat anti-mouse albumin coupled to HRP, Bethyl Laboratories) was added at 1/20000 dilution in 1% skim milk-PBS and the plate was incubate at 37°C for 1 h. Excess of antibody was washed out and TMB substrate (Sigma) was added to develop the enzymatic reaction. After addition of a stop solution (2 N H_2_SO_4_), the absorbance associated with each well was measured at 450 nm in a microplate reader.

Concentration of IgM was measured by ELISA sandwich following the same protocol as described above. Coating was performed with a goat anti-mouse Ig antibody (1/500, Southern Biotech) and the secondary antibody was a goat anti-mouse IgM alkaline phosphatase conjugated (1/4000, Southern Biotech). The enzymatic reaction was developed after addition of alkaline phosphatase substrate (pNPP, Sigma) and measure of absorbance at 405 nm in a microplate reader. Purified mouse IgM (Sigma) was used as standard.

### Bioluminescence measurements

Photon emission of the luminescent bacteria in the lungs of infected mice was measured using the IVIS 100 system (Xenogen Corp., Alameda, CA) as previously described [29]. To achieve detection in C57BL/6 J mice, chest hair was removed with a depilatory agent. 15 h after infection, the mice were anesthetized under 2.5 % isoflurane inhalation and photon emission is acquired by a charge couple device (CCD) camera. A digital false-color photon emission image was generated. The analysis was then performed with the Living Image software (Xenogen Corp., Alameda, CA), by defining a constant region of interested ( ROI) corresponding to the surface of the chest encompassing the whole lung region. The results are expressed as number of photons/sec/ROI.

### Co-exposure of alveolar macrophages to SiO_2_ NPs and *P.a*

Mouse primary AM were isolated after lung bronchoalveolar lavage with PBS [23]. AM (10^5^cells per 96-well plate) were plated in complete RPMI medium (supplemented with 2 mM l-glutamine, 1% antibiotic, and 5% inactivated foetal bovine serum). After 2 h, medium was removed and cells were incubated overnight with fresh medium. The next day, cells were incubated with SiO_2_ NPs (2.5 μg/cm^2^; 0.8 μg/well) in incomplete RPMI medium (without serum or antibiotic) (200 μl) for 5 h. Then a total of 10^5^ cells were infected with bacteria (MOI 0.1 or 0.5). In some wells the same quantity of bacteria were inoculated in the absence of cells to obtain the expected bacterial growth (100%). After 4 h, supernatants (200 μl) were collected and cells were lysed with 0.1% Triton X-100 (a concentration that did not affect PAK viability) in H_2_O in sequential washes to harvest total bacteria. To quantify total viable bacteria, pooled cell supernatants and lysates were diluted and plated on LB agar to determine CFU scores as described previously [32]. Potential cell toxicity after treatment was measured by the CytoTox Non-Radioactive Cytotoxicity Assay (Promega) that measures the percentage of cellular lactate dehydrogenase released.

### Fluorescence microscopy

Primary AM treated or not with FITC-SiO_2_ NPs grown onto coverslips or cytospined cells from BAL were fixed with formalin (10 min room temperature). After blocking (1% BSA-PBS), cells were incubated with rat IgG anti-mouse CD45 antibody (BD Biosciences) followed by Alexa-664-Fluor coupled secondary antibody (Invitrogen) (membrane staining). Phalloidin–tetramethyl-rhodamine (Sigma) was used for labelling cytoplasm. Nuclei were stained with DAPI (Invitrogen). The preparations were mounted with Vectashield (Dako) mounting media and were analysed under structured illumination (ApoTome system, Zeiss) using the corresponding filter for each fluorochrome. Images were recovered and analysed by the AxioVision programme and control cells (not treated with FITC-SiO_2_ NPs) were included to measure fluorescence background. Percentages of positive cells for each preparation were obtained after counting of at least 100 cells in several fields. At least two independent experiments were performed. Representative images are shown in Figures 3 and 7.

### Flow cytometry

BAL fluids from FITC-SiO_2_ NPs treated mice (5 mg/kg) were recovered 5 h post NP instillation and cells were centrifuged at 4°C (2000 rpm, 10 min). Cells were washed in 1% BSA-PBS buffer and incubated with monoclonal anti-mouse CD16/32 antibody (BD Biosciences) for 15 min on ice to block Fc receptors. Cells were then stained with a rat anti-mouse F4/80 antibody coupled to eFluor450 (clone: BM8, eBioscience) and a hamster anti-mouse CD11c antibody coupled to APC (clone: HL3, BD Biosciences) (30 min, at 4°C in 1% BSA-PBS). After washing, fluorescence associated with cells was acquired in a CyAn ADP cytometer (Beckman Coulter). Analysis of data was performed using FlowJo program. FITC positive cells were only found in the macrophage gate (named R1 in FSC/SSC dot plot). To detect FITC positive cells from negative cells, BAL fluids from non-treated mice were obtained and treated in parallel to determine the auto-fluorescence signal (characteristic of AM) from R1 population.

### ALT and CK enzymatic activity in mouse serum

Alanine Aminotransferase (ALT) activity was measured from mouse serum using ALT- activity assay kit (Sigma) following manufacturer’s instructions. The ALT activity is determined by a coupled enzyme assay, which results in a fluorogenic product (λex = 535 nm; λem =587 nm), proportional to the pyruvate generated. ALT activity is reported as nmol/min/mL = mU/mL where one unit (U) is defined as the amount of enzyme that generates 1 μmole of pyruvate per minute at 37°C. Creatine Kinase (CK) activity was measured from mouse serum using CK-activity assay kit (Ray Biotech) following manufacturer’s instructions. The CK activity is determined by a coupled enzyme assay, which results in a colored product with strong optical density at 450 nm. CK activity is reported as nmol/min/mL = mU/mL where one unit (U) of CK is the amount of enzyme that will generate 1.0 μmol of NADH per minute at pH 9.0 at 37°C.

### Statistical analysis

Cytokine levels, polymorphonuclear neutrophil counts, and pathogen counts were expressed as means ± standard errors of the mean (SEM). Differences between SiO_2_ NPs treated and non-treated groups were assessed for statistical significance, using Mann–Whitney test (Prism version 6, GraphPad). Differences were considered statistically significant at p < 0.05. Survival curves were compared with Log-Rank (Mantel-Cox) test (Prism version 6, GraphPad).

## Additional file

Additional file 1: Figure S1. Systemic toxicity of silica nanoparticles during *P.a* infection. Mice were treated with SiO_2_ NPs (5mg/kg; 100μg/mice) or vehicle and 5h later infected with PAK (2.5x10^6^ CFU/mice) (‘SiO_2_/PAK’ and ‘PAK’ groups, respectively). A group of mice were only treated with SiO_2_ NPs (5mg/kg; 100μg/mice) (‘SiO_2_’) and another group only with vehicle (‘vehicle’). 24h or 48h after NP instillation, Alanine Aminotransferase (ALT) and Creatine Kinase (CK) activities were measured in the serum. Mean ± SEM is represented from 5 mice per group.

## Abbreviations

AEC: Alveolar epithelial cells
AM: Alveolar macrophages
ALT: Alanine Aminotransferase
BAL: Broncho-alveolar lavage
CK: Creatine Kinase
MOI: Multiplicity of infection
NPs: Nanoparticles
*P.a*: *Pseudomonas aeruginosa*
SP-C: Surfactant protein C
