# Supplementary figures and images for: Acute exposure to silica nanoparticles enhances mortality and increases lung permeability in a mouse model of Pseudomonas aeruginosa pneumonia
Source: Part Fibre Toxicol. 2015 Jan 21;12(1):1. doi: 10.1186/s12989-014-0078-9 (PMC4318199; doi:10.1186/s12989-014-0078-9)

## Slide 1
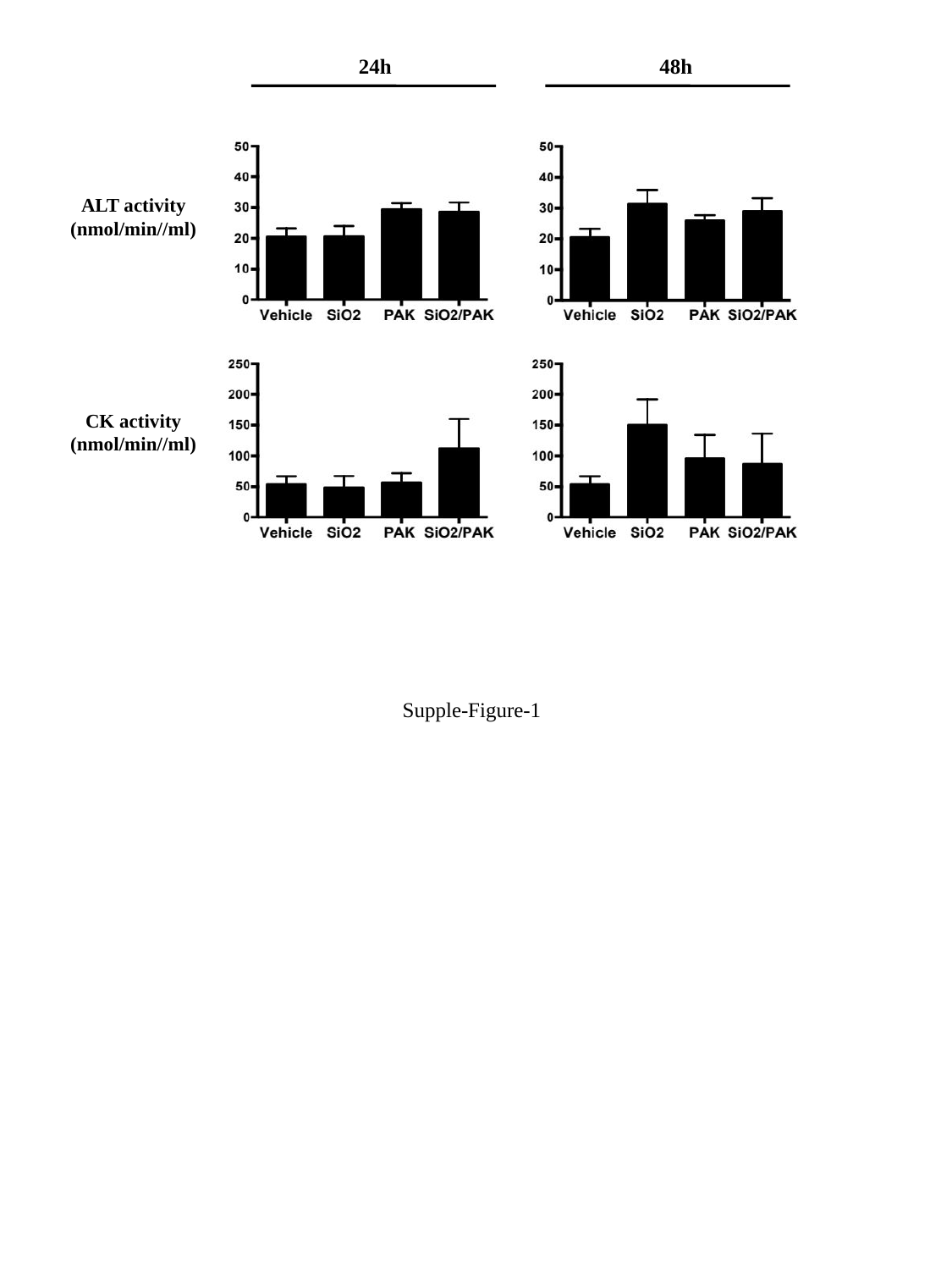

24h
48h
ALT activity
(nmol/min//ml)
CK activity
(nmol/min//ml)
Supple-Figure-1

Supplement: Additional file 1: Figure S1. — Systemic toxicity of silica nanoparticles during P.a infection. Mice were treated with SiO2 NPs (5mg/kg; 100μg/mice) or vehicle and 5h later infected with PAK (2.5x10^6^ CFU/mice) (‘SiO2/PAK’ and ‘PAK’ groups, respectively). A group of mice were only treated with SiO2 NPs (5mg/kg; 100μg/mice) (‘SiO2’) and another group only with vehicle (‘vehicle’). 24h or 48h after NP instillation, Alanine Aminotransferase (ALT) and Creatine Kinase (CK) activities were measured in the serum. Mean ± SEM is represented from 5 mice per group. [file 12989_2014_78_MOESM1_ESM.ppt]
